# Next-generation Janus kinase inhibitors: Integrating synthetic innovation, structural biology, and computational design for precision drug discovery

**DOI:** 10.1016/j.pscia.2026.100116

**Published:** 2026-03-13

**Authors:** Karthik K. Karunakar, Binoy Varghese Cheriyan, Sowmiya Philiph, Rajesh kumar Shanmugam, Josme Sree

**Affiliations:** a Department of Pharmacology, Saveetha College of Pharmacy, Saveetha Institute of Medical and Technical Sciences, Chennai, 602105, Tamil Nadu, India; b Department of Pharmaceutical Chemistry, Saveetha College of Pharmacy, Saveetha Institute of Medical and Technical Sciences, Chennai, 602105, Tamil Nadu, India; c Nanobiomedicine Lab, Department of Anatomy, Saveetha Medical College and Hospital, Saveetha Institute of Medical and Technical Sciences, Chennai, 602105, Tamil Nadu, India

**Keywords:** Janus kinase inhibitors, Structure-based drug design, JAK2/JAK3 selectivity, Computational medicinal chemistry, Signal transducer and activator of transcription (STAT) pathway

## Abstract

Janus kinase (JAK) dysregulation plays a central role in the pathogenesis of inflammatory, autoimmune, and malignant disorders, making the JAK family an essential therapeutic target across multiple disease domains. Over the past two decades, the field has progressed from the identification of early JAK2 inhibitors to the approval of several first-generation agents, including ruxolitinib, tofacitinib, baricitinib, and fedratinib, which validated the clinical feasibility of JAK blockade. However, limitations related to safety, isoform selectivity, long-term tolerability, and off-target kinase interactions continue to restrict their broader application and highlight the need for next-generation molecules. In this review, we provide a comprehensive and strategic assessment of the molecular features underpinning JAK2 and JAK3 selectivity, including signaling features directly relevant to inhibitor design, mutational landscapes, and structural determinants such as the uniquely targetable Cys909 residue in JAK3. Although the JAK family comprises four kinases, this review intentionally focuses on JAK2 and JAK3, where structural divergence, disease relevance, and emerging selectivity strategies provide the strongest opportunities for next-generation precision inhibitor design. We integrate recent advances in synthetic chemistry, including hinge-binding optimization, heterocyclic diversification, multicomponent reactions, and scaffold-hopping strategies, with computational methodologies such as molecular docking, molecular dynamics simulations, QM/MM calculations, and machine-learning-based predictive modelling. Together, these multidisciplinary approaches have accelerated hit discovery, refined selectivity, and improved the pharmacokinetic and safety profiles of emerging JAK inhibitors. By consolidating progress across medicinal chemistry, structural biology, and computational design, this review outlines key opportunities and remaining challenges in developing next-generation JAK inhibitors with enhanced precision and therapeutic value for oncology, immunology, and chronic inflammatory diseases.

## Abbreviations

ADMET: Absorption, Distribution, Metabolism, Excretion, and Toxicity
AI: Artificial Intelligence
ANN: Artificial Neural Network
ATP: Adenosine Triphosphate
CADD: Computer-Aided Drug Design
CV: Cardiovascular
DL, Deep Learning
FEP: Free Energy Perturbation
GNN: Graph Neural Network
HDAC6: Histone Deacetylase 6
IL, Interleukin
JAK: Janus Kinase
MD: Molecular Dynamics
ML, Machine Learning
MM/GBSA: Molecular Mechanics/Generalized Born Surface Area
MPO: Multi-Parameter Optimization
PBPK: Physiologically Based Pharmacokinetic
PK: Pharmacokinetics
QSAR: Quantitative Structure–Activity Relationship;
RA: Rheumatoid Arthritis
RL, Reinforcement Learning
SAR: Structure–Activity Relationship;
SBDD: Structure-Based Drug Design
STAT: Signal Transducer and Activator of Transcription
TYK2: Tyrosine Kinase 2
VTE: Venous Thromboembolism

## 1 Introduction

**Fig. 1:**
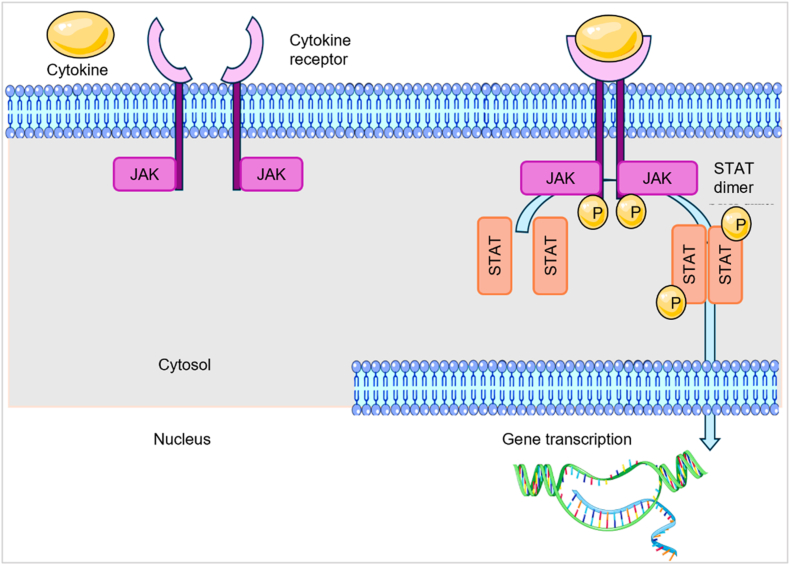
Overview of cytokine-induced activation of the JAK–STAT signaling pathway. Binding of cytokine ligands to their receptors promotes receptor dimerization and activation of associated JAK kinases, leading to reciprocal phosphorylation. Activated JAKs phosphorylate STAT proteins, which then dimerize and translocate to the nucleus to regulate transcription of target genes involved in diverse cellular functions. (produced using biorender).

Inflammatory disorders arise from highly coordinated extracellular and intracellular signalling cascades involving a wide spectrum of cytokines, receptors, kinases, and transcriptional mediators. Although opioids, corticosteroids, and NSAIDs remain the most widely used anti-inflammatory agents, their limited efficacy in chronic conditions and considerable adverse-effect profiles highlight the persistent need for safer and more targeted therapeutic strategies [1,2]. Among intracellular signalling systems, the Janus kinase (JAK)–signal transducer and activator of transcription (STAT) pathway has emerged as a central regulator of cytokine-driven inflammation, hematopoiesis, immune cell maturation, and tumorigenesis [3]. Dysregulated JAK–STAT signalling is strongly implicated in autoimmune diseases, myeloproliferative neoplasms, graft-versus-host disease, and diverse solid tumours [4], [[5], [6], [7]]. JAK kinases—particularly JAK1, JAK2, JAK3, and TYK2—are firmly established as druggable nodes due to their essential roles in cytokine receptor signalling and transcriptional control [8]. First-generation JAK inhibitors such as tofacitinib, ruxolitinib, baricitinib, and fedratinib validated the clinical utility of JAK blockade; however, myelosuppression, infection risk, and off-target kinase interactions reveal the need for inhibitors with improved isoform selectivity and safer long-term profiles [9]. Despite the therapeutic success of early JAK inhibition, clinical experience has exposed fundamental limitations that restrict long-term utility. High sequence and structural homology across JAK isoforms complicate selective ATP-competitive inhibition, often resulting in off-target kinase activity and dose-limiting toxicities. Moreover, sustained pathway suppression is associated with infection risk, hematologic adverse events, and emerging resistance, particularly in oncologic settings. These challenges underscore the need for next-generation JAK inhibitors that achieve precise isoform discrimination, improved safety margins, and durable efficacy through rational structural and mechanistic design. Mechanistically, chronic inflammatory and autoimmune pathways intersect with oncogenic signalling networks, expanding the therapeutic relevance of JAK inhibition and enabling opportunities for multitarget drug development [10]. At the molecular level, JAK proteins comprise conserved FERM–SH2 docking domains, a regulatory JH2 pseudokinase, and the catalytic JH1 tyrosine kinase domain [[11], [12], [13]]. The overall sequence of cytokine-driven JAK activation and STAT-mediated transcription is illustrated in Fig. 1, highlighting the phosphorylation cascade that underpins JAK–STAT signaling.

**Fig. 2:**
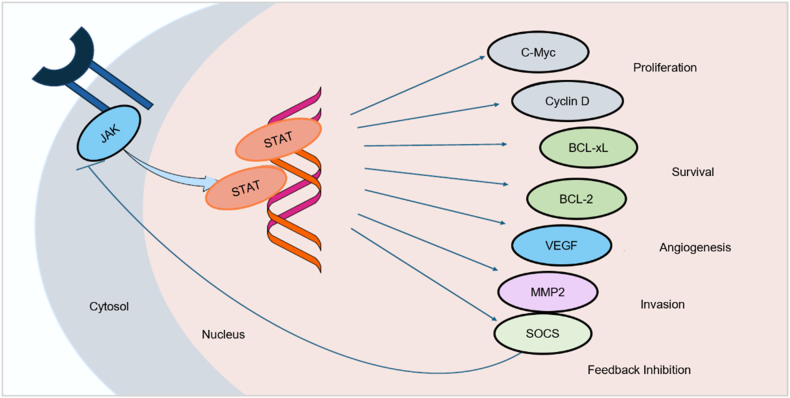
Schematic representation of JAK–STAT signaling and downstream transcriptional responses. Activation of JAK leads to phosphorylation and dimerization of STAT proteins, which translocate to the nucleus and promote transcription of target genes. STAT-driven gene expression regulates key oncogenic processes, including proliferation (C-Myc, Cyclin D), survival (BCL-xL, BCL-2), angiogenesis (VEGF), invasion (MMP2), and negative feedback regulation via SOCS proteins. (produced using biorender).

Cytokine-induced receptor dimerization activates JAKs, resulting in STAT phosphorylation, dimerization, nuclear translocation, and transcription of genes that regulate proliferation, apoptosis, differentiation, and immune homeostasis [10]. To further contextualize these molecular events, Fig. 2 depicts the STAT-driven transcriptional programs that regulate proliferation, survival, angiogenesis, and feedback inhibition. Within this framework, JAK1 and JAK3 coordinate γ-chain cytokine responses—including IL-2, IL-4, IL-7, IL-9, IL-15, and IL-21—governing T-cell activation and survival [14]. JAK1 additionally regulates IL-6/gp130 signalling and CD4^+^ T-cell expansion [15], whereas JAK3 is largely restricted to lymphoid tissues, where it orchestrates lymphocyte maturation and immune function. These divergent yet complementary biological roles continue to guide efforts toward designing selective JAK isoform inhibitors. Addressing these unmet needs has driven a shift from empirical kinase inhibition toward precision-guided drug design integrating structural biology, advanced synthetic chemistry, and computational modeling. Modern JAK inhibitor discovery has transitioned into a multidisciplinary era that integrates synthetic chemistry with advanced computational methodologies [16]. Structure-based drug design (SBDD), ligand-based modelling, molecular dynamics (MD), QM/MM simulations, and free-energy perturbation (FEP) are indispensable for defining ATP-binding site architecture, assessing conformational plasticity, and identifying isoform-specific interaction fingerprints [17]. These tools accelerate hit-to-lead optimization, support scaffold hopping, predict binding energetics, and assist in designing inhibitors that minimise interference from the JH2 pseudokinase domain. Parallel advances in synthetic methodologies—including C–H activation, photoredox catalysis, S_NAr chemistry, multicomponent reactions, fragment-merging, and macrocycle incorporation—have facilitated rapid generation of chemical diversity, hinge-region optimization, and improved scalability in JAK inhibitor synthesis [16]. Clinically, JAK2 and JAK3 have gained substantial attention in hematologic malignancies and myeloproliferative neoplasms, yet selective inhibition of closely related isoforms remains an ongoing challenge. Agents such as ruxolitinib, pacritinib, and AZD1480 exemplify progress in JAK2-targeting strategies [18]. The JAK3 inhibitor landscape continues to evolve, exemplified by ritlecitinib (PF-06651600), which has advanced into late-stage clinical development. In contrast, tofacitinib—initially regarded as JAK3-selective—is highly recognized as a pan-JAK inhibitor, offering broad efficacy but requiring careful safety monitoring. Beyond inflammatory indications, accumulating evidence links JAK–STAT pathway dysregulation to oncogenesis, further expanding the therapeutic relevance of JAK3-targeted strategies [17] and plays critical roles in tissue repair, immune regulation, and hematopoiesis [19]. In this review, we present a strategic evaluation of the synthetic and computational methodologies that have shaped modern JAK inhibitor development. We summarize the evolution of early JAK chemotypes, discuss key advances in synthetic and computational frameworks underlying hit identification and lead optimization, and highlight the convergence of these approaches in rational drug design. Finally, we outline current challenges—including resistance, safety liabilities, and scalability—and provide future perspectives toward next-generation JAK inhibitors with enhanced precision and therapeutic value. Accordingly, this study is intentionally structured around the evolution of JAK2- and JAK3-selective inhibitors, integrating biological rationale, structural determinants, synthetic innovation, and computational design strategies that collectively drive next-generation precision JAK therapeutics.

## 2 Evolution of JAK inhibitors: from pan-JAK blockade to precision selectivity

The historical and mechanistic evolution of JAK inhibition, establishing the clinical and biological context that motivates the transition from first-generation pan-JAK inhibitors to next-generation, isoform-selective strategies. The therapeutic development of Janus kinase (JAK) inhibitors began with the first evidence that JAK2 blockade could suppress leukemic cell proliferation, establishing JAK–STAT signalling as a druggable pathway. Over the following two decades, intensive medicinal chemistry and clinical research translated these early findings into multiple FDA-approved therapies for hematologic, autoimmune, and inflammatory disorders.

### 2.1 Rise of JAK inhibitors

The relevance of JAKs as therapeutic targets emerged in 1996, when a small-molecule JAK2 inhibitor suppressed acute lymphoblastic leukemia (ALL) cell growth [20]. This evidence positioned JAK–STAT signalling as a viable pharmacologic target. In 2011, ruxolitinib—a selective JAK1/2 inhibitor—became the first JAK inhibitor approved by the FDA for intermediate- and high-risk myelofibrosis [21]. Its clinical success spurred further investigation across multiple malignancies, including Hodgkin's disease, supported by early pilot and mechanistic studies [[22], [23], [24], [25]].

Combination approaches soon followed. A Phase 1 trial evaluating ruxolitinib with the BTK inhibitor ibrutinib in chronic lymphocytic leukemia (CLL) demonstrated promising synergistic responses [26]. Ruxolitinib later received a second FDA approval in 2014 for polycythemia vera (PV) [27]. In parallel, tofacitinib—a JAK1/JAK3 inhibitor with moderate activity against JAK2 and TYK2—was approved in 2012 as Xeljanz®, becoming the first oral JAK inhibitor for rheumatoid arthritis (RA). Its therapeutic scope later expanded through Phase 3 trials demonstrating efficacy in ulcerative colitis, chronic plaque psoriasis, alopecia areata, and psoriatic arthritis [16]. Continued progress led to the 2018 approval of baricitinib (Olumiant®), a JAK1/JAK2 inhibitor developed by Eli Lilly, for RA, with Phase 2 studies showing additional promise in diabetic kidney disease, systemic lupus erythematosus, and moderate-to-severe atopic dermatitis [[28], [29], [30], [31], [32]]. Two more JAK inhibitors entered clinical practice in 2019. Fedratinib, a selective JAK2 inhibitor, was approved for high-risk myelofibrosis, while upadacitinib, a JAK1-selective inhibitor initially approved for RA, gained further support from Phase 2/3 trials in ankylosing spondylitis [33,34]. JAK inhibitors are established in many inflammatory and autoimmune diseases, but in oncology ruxolitinib remains the only FDA-approved agent despite widespread JAK dysregulation. JAK2 V617F drives myeloproliferative neoplasms, and activating mutations in JAK1, JAK3, and rare JAK2 variants contribute to T-ALL. JAK1 mutations such as S703I (∼9% of HCC cases) disrupt autoinhibition and amplify signaling. Beyond cancer, JAK1/JAK2 inhibition can reduce HIV-1 reservoirs, and baricitinib has shown clinical benefit in COVID-19 pneumonia by suppressing cytokine-storm inflammation [16].

### 2.2 Targeting JAK2 and JAK3

While early clinical efforts predominantly focused on JAK2 inhibition due to its central role in myeloproliferative disorders, parallel interest in JAK3 rapidly emerged from its restricted expression in hematopoietic cells and its relevance to immune-driven diseases. These early observations established JAK3 as a complementary therapeutic target, laying the clinical and biological groundwork for later structure-guided and covalent JAK3-selective strategies. JAK2 is a ubiquitously expressed intracellular tyrosine kinase essential for erythropoiesis and platelet formation [35]. It transduces signals from cytokines and interferons through gp130-family receptors [36]. Its seven-domain architecture includes the JH1 catalytic kinase domain, the regulatory JH2 pseudokinase domain, the JH3–JH5 receptor-engagement domains, and the N-terminal JH6–JH7 FERM/SH2-like domains responsible for membrane association. Functionally, JAK2 phosphorylates STAT3 at Tyr705, enabling STAT3 dimerization, nuclear translocation, and transcription of inflammatory and oncogenic genes. Pathogenic mutations—especially V617F—drive polycythemia vera, essential thrombocythemia, and myelofibrosis [37]. Pharmacologic JAK2 inhibition induces apoptosis and cell-cycle arrest in preclinical cancer models.

## 3 Biological and structural basis of JAK isoform selectivity

The biological and structural insights that underpin JAK isoform selectivity, focusing on mutation-driven pocket remodeling and non-conserved residues that guide rational inhibitor design. The therapeutic relevance of JAK2 and JAK3 is shaped by their distinct biological roles, structural architectures, and contributions to cytokine-driven immune signalling. Differences in domain organisation, activation mechanisms, mutational profiles, and residue-level selectivity determinants—such as the unique Cys909 in JAK3—form the foundation for isoform-specific inhibitor design. Table 1 provides an overview of major structural scaffolds of JAK inhibitors, highlighting their selectivity profiles and characteristic binding interactions.

**Table 1:** Structural classes of JAK inhibitors and their key interaction features.

Structural class/scaffold	Representative inhibitor	Primary JAK isoform selectivity (clinical/biochemical)	Potency (IC_50_) *approx.*	Key binding residues/interaction features	Inhibition mode (type/covalency)	Reference
2,4-disubstituted pyrimidine (pan-JAK)	**Tofacitinib**	Pan-JAK (JAK1/JAK3 > JAK2; weak TYK2)	JAK3 ∼2 nM; JAK1/JAK2 tens of nM (enzyme)	Hinge binding in ATP pocket; forms H-bonds with conserved hinge residues; engages JAK1/3 more strongly than JAK2/TYK2	Type I, reversible, ATP-competitive	[38]
Imidazopyrimidine (JAK1/2)	**Baricitinib**	JAK1/JAK2-biased inhibitor	Low-nM enzyme IC_50_*vs.* JAK1/2	Forms hinge H-bonds in JAK1; MD shows stable occupancy of ATP site and key hydrophobic contacts	Type I, reversible, ATP-competitive	[39]
Triazole-substituted pyrrolopyrimidine	**Upadacitinib**	JAK1-selective (>40 × vs JAK2; >100 × vs JAK3)	Low-nM JAK1 IC_50_; highly JAK1-biased in cells	Forms key H-bonds with JAK1 hinge residues and optimally fills hydrophobic subpockets, disfavoring JAK2/3	Type I, reversible, ATP-competitive	[40]
Cyanopyridone JAK1 inhibitor	**Abrocitinib**	JAK1-selective	Single-digit nM JAK1 IC_50_ (enzyme)	X-ray shows three H-bonds to Glu957, Leu959, Asn1008 in JAK1 plus hydrophobic contacts with Leu881/Leu959	Type I, reversible, ATP-competitive	[41]
Benzimidazole JAK1/2 inhibitor	**Ruxolitinib**	JAK1/JAK2	Low-nM JAK1/2 enzyme IC_50_	Occupies ATP site of JAK1/2 with canonical hinge H-bonds and hydrophobic packing in back pocket	Type I, reversible, ATP-competitive	[42]
Oxygen-linked macrocycle (JAK2/FLT3)	**Pacritinib (SB1518)**	JAK2/FLT3-biased	Low-nM JAK2 IC_50_	Macrocyclic scaffold wraps around JAK2 ATP site; maintains hinge H-bond network while constraining conformation	Type I, reversible, ATP-competitive, macrocyclic	[43]
1H-Pyrazolo[3,4-d]pyrimidin-4-amino JAK2 series	**Lead compound**	JAK2-selective	JAK2 IC_50_ ∼6–10 nM	Two hinge H-bonds to Leu932; π-stacking with Tyr931; additional H-bonding via acrylamide to Lys857	Type I, reversible, ATP-competitive	[44]
Natural flavonoids/phenolics (virtual and in vitro JAK2 hits)	**Orientin, pulmatin, chlorogenic acid**	Predominantly JAK2 (in silico & enzyme assays)	Predicted low-μM to sub-μM JAK2 IC_50_	Docking shows H-bonding to hinge region (e.g., Leu932) and stabilization via π–π and hydrophobic contacts	Putative Type I, reversible	[45]
Heteroaryl pyrazolone/quinoxalinone dual inhibitors	**Sanachai et al. JAK2/3 hits**	Dual JAK2/3	Sub-μM kinase IC_50_*vs.* JAK2/3	Sulfonamide/heteroaryl motifs form hinge H-bonds (e.g., Glu930, Leu932) and polar contacts with Lys882/Lys905 in JAK2/3	Type I, reversible ATP-competitive	[46]
Pyrazolo[3,4-d]pyrimidine covalent JAK3 chemotype	**Z583**	Highly JAK3-selective (≈4500 × vs other JAKs)	JAK3 IC_50_ ∼0.1 nM	Acrylamide warhead positions for covalent bond to Cys909; maintains hinge H-bonds in JAK3 ATP pocket	Irreversible covalent, Type I pose	[47]

### 3.1 JAK3

JAK3 is a non-receptor tyrosine kinase predominantly expressed in lymphoid tissues, where it pairs with JAK1 to mediate signalling from γc-dependent cytokines, including IL-2, IL-4, IL-7, IL-9, IL-15, and IL-21. This pathway drives T-cell proliferation, B-cell maturation, NK-cell differentiation, and broader adaptive immune responses, making JAK3 central to immune homeostasis. Beyond canonical cytokine signalling, JAK3 interfaces with the PI3K–AKT cascade, supporting cell survival and malignant transformation; constitutive JAK3 activation enhances AKT phosphorylation and promotes pro-survival signalling in hematologic cancers [48]. Pathogenic JAK3 mutations—including R925S, A572V, V722I, and Q988P—drive ligand-independent signalling and heightened STAT5 activation in acute lymphoblastic leukemia (ALL), promoting leukemic proliferation and apoptosis resistance [49]. Genomic profiling broadened the spectrum of JAK3-driven malignancies, implicating aberrant JAK3/STAT activity in anaplastic large cell lymphoma (ALCL) [50], Burkitt lymphoma [51], mantle cell lymphoma, and enteropathy-associated T-cell lymphoma [52]. Emerging data further link gain-of-function JAK3 variants to T-cell prolymphocytic leukemia, cutaneous T-cell lymphoma, and NK/T-cell lymphoma, where they correlate with aggressive disease and IL-2/IL-15-driven immune evasion [53]. A defining molecular feature of JAK3 is the presence of Cys909 within the ATP-binding pocket—a cysteine uniquely not present in JAK1, JAK2, and TYK2. This residue provides a highly exploitable selectivity handle for covalent inhibitor design. Structural studies confirm that Cys909, together with subtle differences in the Met902 gatekeeper and solvent-front residues, creates an extended pocket ideal for covalent and reversible-covalent inhibitor frameworks [54]. Clinically, selective JAK3 targeting is increasingly attractive due to its restricted expression to immune cells, unlike the broadly expressed JAK1, JAK2, and TYK2. Ritlecitinib, a covalent JAK3/Tec inhibitor, has advanced in late-stage clinical development for alopecia areata and vitiligo, with early trials demonstrating durable pathway inhibition and favorable safety profiles [55].

### 3.2 JAK2-selective inhibitors

Rather than representing isolated medicinal chemistry efforts, recent JAK2-directed inhibitors can be broadly categorized by shared scaffold architectures and binding modes that dictate their selectivity profiles. ATP-competitive type I inhibitors based on pyrimidine and pyrazolopyrimidine cores consistently exploit conserved hinge hydrogen bonding, most commonly involving Leu932 and Glu930, which underpins high potency but also limits isoform discrimination due to ATP-pocket homology across JAK family members. In contrast, type II and extended back-pocket binders introduce conformational bias by stabilizing inactive kinase states, yet often suffer from reduced cellular efficacy or suboptimal pharmacokinetics. These trends highlight that while hinge engagement is a universal requirement for JAK2 inhibition, successful selectivity requires additional interactions beyond the conserved ATP-binding region—an insight that frames the comparative studies discussed below.

Pyrazolo[3,4-d] pyrimidine–based inhibitors represent a successful scaffold class for achieving potent and selective JAK2 inhibition. Representative analogues from this series exhibited low-nanomolar activity (IC_50_ ≈ 3 nM) with pronounced selectivity over JAK1 and JAK3, effectively suppressing JAK2/STAT5 signaling in HEL and Ba/F3-JAK2V617F cells and inducing G_0_/G_1_ cell-cycle arrest and apoptosis [56]. 2-Aminopyridine scaffolds have also proven effective for selective JAK2 inhibition, with representative derivatives achieving low-nanomolar potency (IC_50_ ≈ 3 nM) and greater than 70-fold isoform selectivity. Enantiomeric optimization within this scaffold revealed that the R-configuration enhances metabolic stability and reinforces canonical hinge interactions with Leu932 and Glu930, underscoring the importance of stereochemical control for maintaining selectivity [57]. Pharmacophore-guided design of 2-aminopyrimidine scaffolds has enabled the development of dual JAK2/FLT3 inhibitors with attenuated JAK1/3 activity. Representative lead analogues from this class demonstrated potent cellular activity in Molm-13 cells, inducing apoptosis and G_1_/S cell-cycle arrest, consistent with effective JAK2 pathway suppression. Structure-based analyses revealed stable anchoring interactions with key hinge and pocket residues, including Leu932, Val863, Lys943, and Arg980, illustrating how computational modeling informed scaffold optimization and target selectivity [58]. Imidazopyrrolopyridine scaffolds have yielded selective JAK2 inhibitors, with representative analogues showing submicromolar potency (IC_50_ ≈ 0.26 μM), >30-fold isoform selectivity, in vivo suppression of STAT3/STAT5 signaling, and favorable oral bioavailability (∼38%) [59]. Hybrid pyridine–quinoline scaffolds incorporating 2-amino-4-aryl-6-(quinolin-2-ylthio) substitution patterns have emerged as effective JAK2-modulating chemotypes. Representative members of this class suppressed JAK2/STAT3 signaling in MCF-7 breast cancer cells, consistent with direct pathway engagement. Structure-based docking analyses revealed canonical hinge hydrogen bonding with Leu932, complemented by stabilizing hydrophobic interactions involving Leu983, Val863, Ala880, and Met929, highlighting how expanded aromatic frameworks can reinforce JAK2 selectivity through cooperative hinge and pocket interactions [60]. Virtual screening of the JAK2 JH2 pseudokinase domain identified aminoanilinyltriazine scaffolds as allosteric inhibitors, with optimized analogues binding both wild-type and V617F JH2 with micromolar affinity (*K*_d_ ≈ 2–3 μM) through stabilizing hydrogen-bond interactions involving Gln626, Glu627, Val629, and Asn678 [61]. High-throughput virtual screening–driven discovery has proven effective for prioritizing JAK2-binding chemotypes from large chemical libraries. Representative candidates identified from ZINC-based screening exhibited strong predicted binding affinity (docking scores ≈ −10 kcal/mol; MM/GBSA ≈ −46 kcal/mol), with molecular dynamics simulations confirming stable ATP-pocket engagement, sustained hydrogen-bond networks, and low RMSD over extended (100 ns) trajectories. These findings illustrate how integrated docking–MD workflows can reliably enrich for structurally stable JAK2 inhibitors prior to experimental validation [62].

### 3.3 Dual JAK2/3 inhibitors

In contrast to JAK2-focused strategies, selective JAK3 inhibition has been achieved through both scaffold choice and binding-mode innovation. Early non-covalent inhibitors built on indazole, aminopyrimidine, and triazolopyrimidine cores often replicated hinge-binding motifs common to other JAK isoforms, resulting in modest selectivity despite high biochemical potency. More successful approaches emerged from scaffolds capable of exploiting non-conserved features proximal to the ATP pocket, particularly those enabling proximity to the unique Cys909 residue in JAK3. Covalent and reversible-covalent chemotypes, as well as macrocyclic architectures that enforce favorable binding geometry, demonstrated superior isoform discrimination by combining hinge interactions with spatially constrained engagement of JAK3-specific residues. These structure–selectivity relationships explain why certain scaffolds progressed clinically, whereas others failed to achieve meaningful selectivity despite comparable in vitro potency. Pharmacophore-guided screening of pyrazolone-based scaffolds has enabled the identification of dual JAK2/3 inhibitors with balanced isoform activity. Representative analogues from this series displayed effective kinase inhibition across both isoforms, with molecular dynamics simulations revealing that sulfonamide functionalities establish stabilizing hydrogen-bond networks with conserved acidic and basic residues in JAK2 (Glu930/Lys932) and JAK3 (Glu903/Lys905), complemented by favorable van der Waals contacts. These interactions illustrate how rational pharmacophore constraints can be leveraged to achieve dual-isoform engagement while maintaining binding stability [44]. Virtual screening of quinoxalinone scaffolds identified dual JAK2/3 inhibitors with potency comparable to ruxolitinib and tofacitinib, effectively suppressing TF1 and HEL leukemia cell proliferation [63]. Additional JAK2-directed chemotypes have demonstrated effective pathway suppression through direct inhibition of kinase autophosphorylation, leading to apoptosis in cellular models. Molecular dynamics simulations revealed that these inhibitors establish stabilizing hinge-region hydrogen bonds with residues such as Ser936 and Arg938, supporting sustained ATP-pocket engagement and functional inhibition. In parallel, naphthoquinone-based scaffolds, including napabucasin derivatives, have been explored as alternative JAK2-modulating frameworks, further underscoring the structural diversity capable of achieving pathway inhibition through conserved hinge interactions (98) and 2′-methyl napabucasin (99) exerted strong dual JAK2/3 inhibition and outperformed tofacitinib in TF1 and HEL cells [57]. These inhibitors induced apoptosis and engaged conserved hinge residues (Tyr931 and Leu932), supported by hydrophobic interactions spanning the hinge, G-loop, and catalytic loop of JAK2, underscoring their potential as anticancer JAK-targeted leads [60].

### 3.4 Naturally derived JAK inhibitors

Here, synthetic strategies are critically evaluated in the context of how specific chemotypes and reaction platforms enable access to structurally and functionally selective JAK inhibitors. Natural-product–derived chemotypes have also been explored as modulators of JAK3 signaling. Representative herbal scaffolds demonstrated micromolar inhibition of JAK3 activity and effectively suppressed IL-2–induced JAK3 phosphorylation in HEK293 cells, indicating functional pathway engagement. Structure-based docking analyses revealed that select analogues establish hydrogen-bond interactions with key active-site residues, including Ala966, Asp967, Glu903, Leu905, and the JAK3-specific Cys909, whereas other members of the series achieved comparable binding affinity through alternative non–hydrogen-bonding interactions. Molecular dynamics simulations further confirmed stable ligand–JAK3 complexes, with low RMSD values (≈1.5–3 Å), underscoring the capacity of structurally diverse natural scaffolds to engage the JAK3 active site [64]. Insect-derived metabolites isolated from *Blaps japanensis* have yielded potent cytotoxic chemotypes active against A549, Huh-7, and K562 cells, with demonstrated JAK3 inhibitory activity, highlighting the pharmacological diversity of insect secondary metabolites [65]. Structure-based design has enabled the development of highly selective JAK3 inhibitors with robust cellular activity. Representative chemotypes identified through docking-driven optimization exhibited strong predicted binding affinity (docking scores ≈ −12 kcal/mol) and engaged a network of active-site residues spanning both hinge and hydrophobic regions, including Val812, Ala829, Glu847, Met878, Leu881, Leu932, and Asp943. These inhibitors achieved low-nanomolar JAK3 potency (IC_50_ ≈ 10 nM) with minimal cross-reactivity toward JAK1, JAK2, and TYK2, translating into pronounced induction of apoptosis and autophagic/necrotic cell death across multiple cancer cell models [66]. Collectively, these structural and biological insights reveal that JAK isoform selectivity is governed less by gross architectural differences and more by subtle non-conserved residues, mutation-induced pocket remodeling, and regulatory-domain conformational dynamics. While conserved hinge interactions are necessary for potency, they are insufficient for selectivity, underscoring the importance of exploiting peripheral pockets, pseudokinase (JH2) regulation, and unique residues such as Cys909 in JAK3. These principles provide the mechanistic foundation for the synthetic and computational strategies.

## 4 Synthetic innovation and structure–activity relationships

Our current study integrates structure-based and AI-driven computational approaches as enabling tools that guide scaffold selection, synthetic decision-making, and selectivity optimization. Importantly, recent synthetic innovations in JAK inhibitor discovery have increasingly been guided by structure-based and computational insights rather than pursued in isolation. In several cases, in silico identification of isoform-specific binding determinants—such as proximity to the JAK3 Cys909 residue or access to extended back-pocket regions—has directly informed the choice of synthetic strategy, including scaffold hopping, late-stage C–H functionalization, and electrophile installation. These computationally driven design hypotheses necessitated flexible and modular synthetic routes capable of rapidly incorporating predicted warheads, stereochemical features, or conformational constraints, thereby tightly coupling synthetic feasibility with structure-guided optimization. Recent advances in medicinal chemistry have enabled the development of diverse small-molecule scaffolds targeting JAK, offering new opportunities to optimize potency, selectivity, and anticancer activity. Structure–activity relationship (SAR) studies across multiple chemotypes show how subtle changes in heterocycles, linkers, and functional groups influence cellular response and kinase inhibition. Complementary molecular docking analyses illuminate key interactions within the ATP-binding pocket, guiding the rational design of next-generation JAK inhibitors. Importantly, the choice of synthetic strategy in JAK inhibitor development is not interchangeable: early-stage scaffold exploration favors modular and diversity-oriented approaches, whereas late-stage lead optimization increasingly relies on methods that enable precise functional-group placement, stereochemical control, and scalability. Distinguishing these application scenarios is essential for understanding how synthetic innovation directly supports structure–activity relationship refinement and translational progression.

Hybrid anticancer strategies are gaining momentum, as exemplified by heterosteroid-based scaffolds that exhibit potent cytotoxicity against HepG2, Huh-7, and A549 cells. Structure-based docking predicted strong JAK2 binding affinity (≈−11 to −12 kcal/mol), driven by hydrophobic interactions with key residues such as Leu932, Leu983, and Val863, alongside stabilizing hydrogen-bond contacts within the hinge region. These findings suggest that the steroidal framework contributes significantly to enhanced binding stability and anticancer activity [1]. Pharmacophore merging of known drugs has enabled the development of 4-piperazinyl-2-aminopyrimidine scaffolds with potent JAK2 inhibitory activity. Representative analogues displayed strong antiproliferative effects in HEL, MV4-11, and HL60 cells, inducing dose-dependent apoptosis and G1/S cell-cycle arrest. Docking analyses showed that the 2-aminopyrimidine core occupies the JAK2 ATP pocket via dual hinge hydrogen bonds to Leu932, supported by alkyl–π interactions with Ala880, Leu855, Val863, and Leu983, along with additional stabilizing contacts involving Lys943 and Leu855 [56]. From a strategic perspective, multicomponent reactions and scaffold-hopping approaches are particularly valuable for rapidly generating chemical diversity during hit identification, whereas C–H activation and S_NAr chemistry enable late-stage functionalization critical for tuning hinge-binding interactions and isoform selectivity. Macrocyclization and hybrid scaffold design, although synthetically more demanding, are preferentially applied when conformational restriction or dual-target engagement is required, especially in advanced lead optimization. Recent medicinal chemistry has produced diverse JAK2/JAK3 inhibitors, with SAR and computational studies clarifying how small structural changes drive potency and selectivity. 3,5-Disubstituted pyrazole–pyrazolopyrimidine scaffolds have demonstrated strong JAK2 inhibitory activity, with representative analogues achieving low-nanomolar potency (IC_50_ ≈ 6–10 nM) and exhibiting cell-line–dependent efficacy in K562 and HEL models. Structure–activity trends indicated that meta-substitution enhanced binding affinity, while docking analyses revealed conserved hinge hydrogen bonding with Leu932, π–π stacking with Tyr931, and stabilizing hydrophobic and Lys857-mediated water-bridge interactions that collectively support high potency [44]. 2,4-Disubstituted quinazoline scaffolds have been explored as cytotoxic JAK2-modulating chemotypes, with representative analogues exhibiting micromolar antiproliferative activity (IC_50_ ≈ 10 μM) and moderate inhibition of JAK2 enzymatic activity. Structure–activity analysis indicated that hydrophobic aromatic substitution combined with hydroxyl or nitro functionalities enhanced biological activity. Structure-based docking revealed stronger predicted binding for the optimized analogue (binding energy ≈ −8.6 kcal/mol) relative to closely related variants (≈−7.7 kcal/mol), with both engaging canonical hinge hydrogen bonds to Leu932 and additional contacts with Arg938 and Asp994. Molecular dynamics simulations further demonstrated greater complex stability for the optimized scaffold (RMSD <1 Å) compared with more conformationally flexible analogues (RMSD >2 Å), highlighting how subtle structural modifications can markedly influence binding stability and functional activity [63]. Other studies combining rational design, biological assays, and docking identified two highly selective JAK3 inhibitors. Docking highlighted Cys909 as the key selectivity determinant, with aryl groups positioned to form covalent bonds with its sulfur—an interaction absent in JAK1/2/TYK2 and central to covalent JAK3 inhibitor design [67,68]. S-adenosylmethionine (SAM) showed strong anticancer activity in gallbladder cancer cells, suppressing proliferation, inducing apoptosis and G_0_/G_1_ arrest. SAM reduced p-JAK2, and dual JAK2/STAT3 blockade enhanced apoptosis. *In vivo*, SAM lowered tumor mass and p-JAK2 levels, confirming it as a natural JAK2/STAT3 modulator [69]. 2-Aminopyridine scaffolds have yielded highly potent and selective JAK2 inhibitors, with enantiomeric resolution revealing the R-configuration as the active form. Representative analogues achieved low-nanomolar potency (IC_50_ ≈ 3.0 nM) with strong selectivity over JAK1 and JAK3, supported by molecular dynamics simulations showing stable ATP-pocket binding and canonical hinge interactions with Leu932 and Glu930. Structure–activity analysis further indicated that oxygen-containing ring substituents contribute critically to enhanced potency [57]. 2-Aminopyrimidine scaffolds have produced potent dual JAK2/FLT3 inhibitors with selective antiproliferative activity in HEL and Molm-13 cells, while showing weaker effects in K562 and PC-3 models. Representative analogues induced apoptosis, triggered G_1_/S cell-cycle arrest, and displayed moderate metabolic stability (*t*_½_ ≈ 31 min). Docking analyses revealed canonical hinge binding to Leu932, complemented by hydrophobic interactions with Val863, Ala880, Val911, Met929, and Leu983, as well as stabilizing hydrogen bonds involving Lys943, Asp976, and Arg980 [58]. 2-Amino-4-aryl-6-(quinolin-2-ylthio)pyridine-3,5-dicarbonitrile scaffolds have shown notable cytotoxic activity in MCF-7 and A549 cells, with representative analogues effectively downregulating JAK2/STAT3 signaling in breast cancer models. Docking analyses revealed a canonical hinge hydrogen bond to Leu932, supported by extensive lipophilic interactions with residues including Leu983, Leu855, Val863, Pro933, Met929, and Ala880, establishing a clear mechanistic link between binding mode and biological activity [60]. Imidazopyrrolopyridine scaffolds have yielded selective JAK2 inhibitors with submicromolar potency (IC_50_ ≈ 0.26 μM) and greater than 30-fold isoform selectivity. In GM-CSF–treated mouse models, representative analogues suppressed STAT3/STAT5 phosphorylation to baseline levels and demonstrated favorable pharmacokinetics, including ∼38% oral bioavailability, a 1.9-h half-life, and good metabolic stability. Docking analyses revealed strong hinge hydrogen bonding to Glu930 and Leu932, along with a cyano–Lys882 interaction critical for JAK2 specificity [59].

Virtual screening of the JAK2 JH2 pseudokinase domain identified an aminoanilinyltriazine scaffold as an allosteric inhibitor series. Structural analyses revealed stabilizing hydrogen-bond interactions with residues such as Gln626, Glu627, and Val629, while subsequent optimization produced analogues with micromolar affinity (K_d_ ≈ 2–3 μM) toward both wild-type and V617F JH2. Docking further highlighted interactions with Gln626, Glu628, Val629, Asn678, Asn673, Thr555, and Arg715, consistent with stabilization of the JH2 regulatory conformation and effective allosteric modulation [61] High-throughput virtual screening of the ZINC database identified a JAK2-binding chemotype with predicted affinity comparable to ruxolitinib, supported by strong docking (≈−10.1 kcal/mol) and MM/GBSA (≈−46.1 kcal/mol) scores. The ligand engaged key hydrophobic residues (Leu855, Val863, and Leu983) and formed canonical hinge hydrogen bonds with Glu930 and Leu932. Molecular dynamics simulations confirmed a stable protein–ligand complex over 100 ns, with low RMSD (≈1.7 Å for the protein and 0.4 Å for the ligand) and a persistent network of 3–4 hydrogen bonds [62]. Collectively, these SAR and docking studies reveal consistent structural motifs—such as Leu932 hinge binding, hydrophobic anchoring in the Val863/Leu983 region, and selective targeting of unique amino acids like Cys909—that govern potency and isoform selectivity. Together, these insights provide a predictive blueprint for rational JAK inhibitor design and underscore how synthetic chemistry and computational modeling continue to accelerate the development of next-generation JAK-targeted therapeutics.

## 5 Enhancing JAKis discovery through structure-based drug design strategies

The development of new drugs requires substantial time and financial investment, with recent estimates indicating a doubling of overall costs. Advances in information technology have introduced a wide array of cheminformatic and bioinformatic tools, making computer-aided drug design (CADD) an indispensable component of modern drug discovery in both academic and industrial settings. Parallel progress in structural techniques, including X-ray crystallography and NMR, has accelerated the rise of structure-based drug design (SBDD), which leverages crystallographic data to define ligand-binding modes and guide the creation of high-affinity molecules; SBDD is a leading strategy across multiple therapeutic areas [16].

**Fig. 3:**
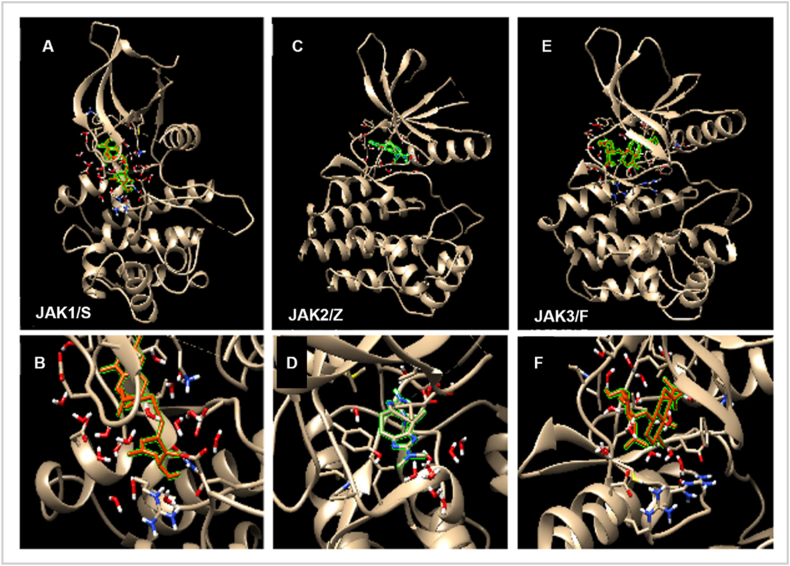
Structural, Docking, and Molecular Dynamics Overview of JAK Isoforms and Lead. Comparative docking conformations across JAK1, JAK2, and JAK3, illustrating ligand orientation and major hydrogen-bond and hydrophobic contacts within the ATP pocket. Reproduced from Ref. [73], under the terms of the Creative Commons Attribution License (CC BY 4.0), (2024) MDPI (Multidisciplinary Digital Publishing Institute).

The availability of crystal structures for all JAK isoforms has enabled extensive SBDD-driven efforts in the design of JAK inhibitors. For example, the structure-based development of (benz)imidazole pyridones produced a JAK1-selective hit and identified Arg879 and Glu966 as key residues governing JAK1 selectivity [70]. Further advances came from a fragment-based approach by Hansen et al., who expanded a 6-arylindazole series and, using crystallographic information, developed a pyrazolopyridone inhibitor with nanomolar potency and strong JAK1 selectivity [71]. Three studies from two Chinese groups developed JAK3-selective inhibitors—across pyrazolopyrimidines, pyrimidine-4,6-diamines, and phenyl-pyrimidines—using the same workflow of derivative design, in vitro testing, and docking. Highly selective JAK3-binding chemotypes were identified, with docking analyses highlighting Cys909 as the critical determinant of isoform selectivity and supporting a covalent interaction between the aryl moiety and the Cys909 sulfur atom [67]. Additional work by Bajusz et al. established a validated SBDD-based in silico protocol to discriminate actives from decoys and identify JAK2-over-JAK1 selective hits, eventually leading to the discovery of an indazole inhibitor with 14-fold selectivity and later an optimized derivative—3-amino-N-(2,6-dichlorophenyl)-1H-indazole-5-carboxamide—with nanomolar JAK2 potency and 16-fold specificity [72]. Notably, these studies also underscored the critical role of structured water networks within the JAK2 binding site, emphasizing their importance in computational drug design. Fig. 3 presents representative docking poses, domain architectures, and MD-derived stability metrics across JAK isoforms, highlighting conserved and isoform-specific binding determinants.

## 6 Computational design strategies for JAK inhibitors: addressing key design challenges

Computational methodologies have become indispensable in modern JAK inhibitor discovery, enabling the rational navigation of complex structure–activity landscapes, isoform selectivity engineering, and efficient prioritization of synthetically tractable chemotypes. Rather than functioning as isolated techniques, contemporary machine learning (ML), artificial intelligence (AI), and physics-based modeling approaches are best viewed as complementary tools, each tailored to address specific design challenges encountered across hit identification, lead optimization, and translational refinement.

### 6.1 Design challenge: large-scale SAR learning and scaffold prioritization

A central challenge in JAK inhibitor discovery is the extraction of robust structure–activity relationships (SAR) from chemically diverse datasets spanning multiple isoforms (JAK1/2/3/TYK2). ML and AI approaches have proven particularly effective in addressing this challenge by learning non-linear SAR patterns and enabling rapid prioritization of selective chemotypes. Importantly, different ML architectures contribute distinct strengths rather than serving interchangeable roles.

Graph neural networks (GNNs), which operate directly on molecular graph representations, excel at capturing subtle substructure–selectivity relationships across closely related JAK isoforms. Multitask GNN-based QSAR models have been developed to simultaneously predict IC_50_ values for JAK1–3, providing both high predictive accuracy and interpretable feature attributions that highlight substructures driving potency and isoform bias [74], (Table 2A). Such models are particularly well suited for scaffold optimization, where fine-grained chemical modifications dictate selectivity outcomes. In contrast, descriptor-based ensemble methods such as extreme gradient boosting (XGBoost) rely on engineered molecular fingerprints and demonstrate superior performance in rapid potency ranking, ADMET-aware triaging, and large-library filtering, where computational speed, robustness, and interpretability are prioritized [75]. Hybrid QSAR–ANN frameworks have further shown strong validation performance for JAK1/JAK3-selective heterocycles, reliably prioritizing active scaffolds prior to synthesis and thereby reducing experimental burden [76]. Beyond single-target modeling, ML pipelines have been extended to polypharmacology challenges. Dual-target discovery workflows—for example, those targeting JAK2/HDAC6—combine k-nearest neighbours, gradient boosting, and LightGBM classifiers with SHAP-based interpretability to identify molecular fragments contributing to balanced multi-target activity [77]. Generative deep-learning models have further expanded accessible chemical space by enabling automated scaffold innovation. Graph-based variational autoencoders and scaffold-linking frameworks such as GraphGMVAE and SyntaLinker-Hybrid recombine validated hinge-binding motifs with novel molecular cores, producing kinase-like scaffolds that retain JAK binding determinants while exploring new regions of chemical space [78]. More broadly, multi-view scaffold generators such as ScaffoldGVAE explicitly incorporate scaffold constraints, an essential consideration for kinases where binding geometry strongly governs activity and selectivity [79]. Reinforcement learning–based generative workflows have also enabled macrocycle design—historically challenging for kinase inhibitors—yielding macrocyclic fedratinib analogues with improved predicted potency and physicochemical profiles [80]. AI-assisted repurposing approaches further illustrate the translational utility of these tools, with deep-learning models trained on FDA-approved drugs successfully identifying existing compounds as potential JAK2 inhibitors, subsequently validated through docking, molecular dynamics, and biological assays [81]. Despite their power, ML-based approaches remain dependent on data quality and chemical-space representation, and their extrapolative capacity beyond well-sampled chemotypes can be limited. Consequently, ML-driven prioritization is most effective when integrated with structure-based and physics-informed validation strategies.

**Table.2A:** Computational design strategies for JAK inhibitor discovery categorized by design objective.

Design objective	Primary computational strategy	Representative techniques/algorithms	JAK isoform focus	Key outcome for JAK inhibitor design	Reference
Large-scale SAR learning and potency prediction	Machine-learning QSAR	Multitask GNN, XGBoost, RF, ANN	JAK1/2/3/TYK2	Enables rapid prediction of pIC_50_ values and identification of substructures driving isoform selectivity.	[[91], [92], [93]]
Scaffold prioritization and virtual screening	Hybrid ML + ligand-based models	QSAR, ECFP4, ensemble ML	JAK1/2/3	Efficient ranking and triaging of large chemical libraries prior to docking or synthesis	[[92], [93], [94]]
Binding-mode validation	Structure-based modeling	Molecular docking, MD simulations	JAK2, JAK3	Identification of stable ATP-pocket and back-pocket interactions governing potency and selectivity	[76,82]
Isoform selectivity engineering	Physics-based free-energy calculations	MM/PBSA, FEP+, QM/MM	JAK2, JAK3	Quantitative discrimination of closely related JAK isoforms and energetic basis of selectivity	[83,90]
Covalent inhibitor optimization	Covalent docking and reactivity modeling	Covalent docking, QM/MM	JAK3	Precise tuning of electrophilic warheads targeting Cys909 for durable and selective inhibition	[90]
Macrocycle and scaffold innovation	Generative AI models	CycleGPT, Macformer, graph-based VAE	JAK2	Exploration of constrained macrocyclic chemical space with improved potency and selectivity	[80]
Polypharmacology and dual-target design	Interpretable ML + MD	RF/XGB + SHAP + MD	JAK2/JAK3 (±HDAC6, BTK)	Rational identification of dual-target inhibitors and feature attribution for balanced activity	[77]
Safety and developability screening	*In silico* ADMET prediction	SwissADME, pkCSM, ProTox-II	Pan-JAK	Early elimination of scaffolds with CYP, hERG, or solubility liabilities	[95]
Drug repurposing and indication expansion	Network-based AI	Knowledge-graph and network models	JAK1/JAK2	Identification of new therapeutic indications for approved JAK inhibitors	[96]

### 6.2 Design challenge: Binding-mode optimization and isoform selectivity engineering

While ML excels at SAR scaling and prioritization, achieving precise isoform selectivity requires detailed understanding of ligand–protein interactions and binding energetics. Structure-based computational tools address this challenge by resolving binding modes, conformational dynamics, and energetic contributions that underpin selective inhibition.

Multi-tiered in silico pipelines integrating pharmacophore modelling, molecular docking, molecular dynamics (MD) simulations, and MM/PBSA calculations have been widely applied to JAK2 and JAK3 inhibitor discovery. These workflows enable pre-synthetic thermodynamic filtering and identification of stable, functionally relevant binding poses, particularly in the conserved ATP-binding pocket [82]. Free-energy perturbation (FEP+) approaches have further been used to balance TYK2 potency against selectivity over JAK2 and JAK3, providing a transferable framework for engineering JAK-family selectivity through quantitative energetic comparisons [83]. Table 2b collectively summarize how these structure-based and ML-assisted tools are deployed to resolve binding determinants, validate predicted interactions, and prioritize candidates with favorable selectivity profiles. Nevertheless, docking and MD simulations are sensitive to protein conformation selection and scoring-function limitations, while free-energy methods remain computationally demanding. As such, these approaches are most effective when applied selectively to high-confidence candidates emerging from ML-guided triage rather than as primary large-scale screening tools.

**Table.2b:** Representative AI- and computational-driven studies in JAK inhibitor discovery.

Computational approach	Specific method/model	Primary application	JAK isoform(s)	Key contribution	Reference
Multitask ML-QSAR	Interpretable GNN regression	Potency and selectivity prediction	JAK1/2/3/TYK2	Single multitask model predicting pIC_50_ across all JAKs and highlighting selectivity-driving substructures	[91]
Ensemble ML	CoGT (XGBoost-based ensemble)	Isoform inhibition classification	JAK family	Improved robustness and generalization compared with single ML models	[92]
Descriptor-based ML	XGBoost regression QSAR	Early virtual screening	JAK2	Fast, accurate pIC_50_ prediction for large libraries	[93]
Hybrid ML + structure	DNN-QSAR + pharmacophore + docking + MD	Hit identification from ZINC	JAK1	Identified novel JAK1 inhibitors with confirmed in vitro activity	[94]
Interpretable ML + MD	RF/ET/XGB + SHAP + MD	Dual-target discovery	JAK3 + BTK	Identified dual BTK/JAK3 inhibitor and rationalized selectivity features	[97]
Geometric deep learning	DeepDock + docking + MD	Virtual screening	JAK3	Discovered structurally novel JAK3 inhibitor beyond classical kinase scaffolds	[98]
Integrated CADD pipeline	QSAR + docking + MD	Selective inhibitor profiling	JAK1/JAK3	End-to-end workflow guiding synthesis and selectivity optimization	[76]
Polypharmacology ML	KNN, GBDT, LGBM + SHAP	Dual-target prediction	JAK2 + HDAC6	High-accuracy classifiers revealing key fragments for dual inhibition	[77]
Generative AI (macrocycles)	Macformer	Macrocyclization	JAK2	Automated generation of macrocyclic JAK2 inhibitors	[80]
Transformer-based AI	CycleGPT + docking	Prospective macrocycle design	JAK2	Designed ultra-potent macrocyclic inhibitors (IC_50_ ≈ 1–2 nM)	[99]
Kinome-wide ML benchmark	Comparative ML/DL evaluation	Kinase inhibition profiling	JAKs among 354 kinases	Established best practices for kinase-focused ML modeling	[100]
Network-based AI	Knowledge-graph analysis	Drug repurposing	JAK1/JAK2	Identified baricitinib as a candidate for COVID-19 therapy	[96]

### 6.3 Design challenge: Engineering durable target Engagement and safety profiles

Beyond potency and selectivity, a critical challenge in JAK inhibitor development is achieving durable target engagement while minimizing systemic toxicity. Computational design strategies play a pivotal role in guiding both non-covalent and covalent inhibitor development to address this balance.

**Fig. 4:**
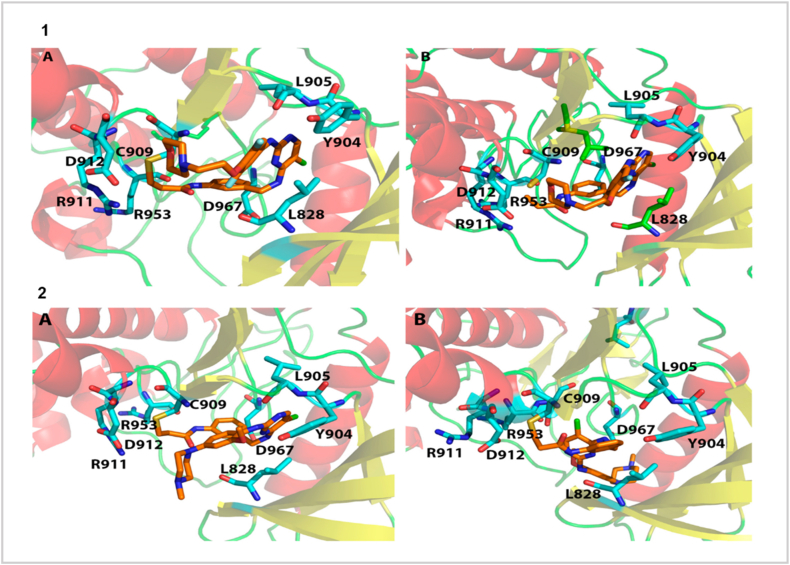
Structural comparison of JAK inhibitor binding modes. **(A)** Docking poses of the inhibitor within the JAK3 active site, showing conserved hinge interactions and proximity of the electrophilic warhead to Cys909, supported by contacts with L828, L905, Y904, D912, R911, and R953. **(B)** Alternative binding overlays highlighting subtle ligand shifts while maintaining key hydrogen bonds and hydrophobic interactions that drive JAK3 selectivity. Reproduced from Ref. [101], under the terms of the **Creative Commons Attribution License (CC BY 4.0) International Journal of Molecular Sciences (IJMS)**.

Traditional non-covalent ATP-competitive JAK inhibitors achieve isoform bias through hinge and back-pocket interactions but require sustained systemic exposure, which has been associated with class-related adverse events such as serious infections, thromboembolic events, malignancy risk, and major adverse cardiovascular events [84]. In contrast, covalent JAK3 inhibitors exploit the uniquely positioned Cys909 residue (Ser in JAK1/2/TYK2), enabling irreversible or slowly reversible binding with intrinsic isoform selectivity [85]. Early 2,4-pyrimidine and cyanamide chemotypes demonstrated sub-nanomolar potency and >100-fold JAK3 selectivity through well-positioned electrophilic warheads, supported by co-crystal structures and docking/MD analyses [86]. This strategy culminated in ritlecitinib (PF-06651600), whose acrylamide warhead engages Cys909 to deliver durable target occupancy and clinical efficacy in alopecia areata [87]. To mitigate off-target liabilities, reversible covalent warheads such as cyanoacrylamides and tuned nitriles have been developed to balance residence time with controlled reactivity [88]. Kinase-wide computational assessments now emphasize precise tuning of electrophile geometry and reactivity to favor the desired cysteine while minimizing proteome-wide engagement—principles central to next-generation JAK3 discovery [89]. Emerging workflows integrate covalent docking, QM/MM calculations, and kinome-wide off-target prediction to optimize warhead placement and isoform specificity beyond what is achievable with non-covalent scaffolds alone [90]. Clinically, these binding-mode differences translate into distinct safety profiles: selective JAK2 inhibition is commonly associated with hematologic toxicities, whereas covalent JAK3 inhibitors aim to preserve efficacy while reducing myelosuppression due to the lymphoid-restricted expression of JAK3. Collectively, these computational strategies demonstrate how binding mode, residence time, and isoform selectivity can be rationally engineered to directly shape clinical tolerability and therapeutic durability. Fig. 4 illustrates structural comparison of JAK inhibitor binding modes.

## 7 ADMET and pharmacokinetic optimization of JAK inhibitors

ADMET and PK optimization are central to oral JAK inhibitor design, requiring rapid absorption, adequate exposure, and predictable clearance across inflammatory and oncologic settings [102]. Tofacitinib shows high oral bioavailability but relies on CYP3A4 (and minor CYP2C19) metabolism, making its exposure highly sensitive to azoles, ritonavir, and other CYP3A4 modulators [103,104]. Baricitinib is primarily renally excreted as unchanged drug (∼70%–75%), so renal function is the major driver of exposure and dictates eGFR-adjusted dosing [105]. Upadacitinib extended-release demonstrates dose-proportional PK, ∼80% bioavailability, and a 2–4 h t_max, but shares CYP3A4-mediated drug–drug interaction risks relevant in polypharmacy common to rheumatology and dermatology [102,106]. PBPK and population PK models for tofacitinib and baricitinib increasingly guide exposure predictions in pediatrics, organ impairment, and co-medication scenarios to support precision dosing [107,108]. Design-wise, tuning cLogP, solubility, and permeability remains essential: highly lipophilic JAK scaffolds often show low solubility, high protein binding, and increased hERG/off-target kinase risk, prompting recent JAK1/JAK3 programs to prioritize moderate lipophilicity and high predicted GI absorption early in SAR [76]. In silico ADMET tools such as SwissADME, pkCSM, and ProTox-II are standard in JAK inhibitor discovery, screening virtual libraries for drug-likeness, permeability, CYP liability, and cardiotoxicity; recent JAK3 covalent and multi-kinase studies explicitly applied these filters to identify scaffolds with balanced solubility–permeability before synthesis [95,109]. To facilitate comparison across JAK inhibitors, their pharmacokinetic and ADMET profiles can be broadly differentiated along three clinically relevant axes: metabolism, excretion, and drug–drug interaction risk. JAK1- and JAK3-selective inhibitors are predominantly cleared via hepatic metabolism, often involving CYP3A4, which increases susceptibility to drug–drug interactions with strong CYP inhibitors or inducers. In contrast, JAK2-biased inhibitors frequently display mixed hepatic and renal clearance, reflecting the broader tissue distribution and systemic roles of JAK2, and are more commonly associated with hematologic exposure-dependent liabilities. Differences in plasma protein binding, half-life, and oral bioavailability further influence dosing frequency and exposure margins. Framing ADMET properties around these core determinants clarifies how pharmacokinetic behavior intersects with isoform selectivity, safety, and clinical usability.

### 7.1 Pharmacokinetic profiles and clinical determinants of JAK inhibitor exposure

#### 7.1.1 Oral absorption and bioavailability

The first key determinant of systemic exposure for oral JAK inhibitors is efficient gastrointestinal absorption and adequate bioavailability. Tofacitinib shows ∼74% absolute oral bioavailability with rapid absorption (*t*_max_ ≈ 0.5–1 h) in healthy subjects, supporting once- or twice-daily dosing [110]. Recent PBPK work confirms that even modest reductions in bioavailability can meaningfully alter steady-state exposure for JAK inhibitors with narrow safety margins [108]. Hepatic CYP metabolism is a second major driver of exposure and DDI risk: tofacitinib is predominantly metabolized by CYP3A4 with a minor CYP2C19 contribution, so strong CYP3A4 inhibitors (e.g., azoles, ritonavir-boosted regimens) or inducers (e.g., rifampin) can markedly change exposure and require dose adjustment [111,112]. Upadacitinib extended-release shows dose-proportional PK and ∼80% bioavailability, but similarly relies on CYP3A4 and thus shares DDI concerns in poly-medicated patients [113]. By contrast, baricitinib is cleared largely as unchanged drug in urine (∼70%–75% of dose), making renal function the dominant determinant of exposure [114]. Population PK analyses in adults and children consistently identify renal function and body size as key covariates of baricitinib clearance [115]. PBPK simulations in geriatric patients with chronic renal impairment suggest that standard 4 mg dosing can yield ∼2.3-fold higher exposure in some older subgroups, supporting dose reduction [116]. For tofacitinib and baricitinib, integrated PBPK/PopPK frameworks are highly routinely used to bridge across renal/hepatic impairment, paediatrics, and elderly populations, and to quantify transporter-mediated interactions such as OAT3 inhibition [117,118].

### 7.2 In silico ADMET screening and physicochemical optimization strategies

Optimizing physicochemical and ADMET profiles is highly essential in JAK inhibitor design, as early scaffolds showed high lipophilicity, poor solubility, and off-target or hERG liabilities. Modern workflows incorporate computational ADMET filters, permeability and solubility prediction, and early toxicity screens to reduce attrition. Lipophilicity is a key driver: overly lipophilic chemotypes show low solubility, high protein binding, and greater metabolic load, whereas maintaining moderate cLogP (2–3) with added polarity improves absorption and lowers hERG risk, as shown in recent ADMET-guided JAK1/JAK3 programs [119]. Early solubility/permeability triaging using BOILED-Egg, BCS models, and GI-absorption predictions are standard. A multikinase-targeting JAK inhibitor study used SwissADME to eliminate scaffolds with predicted low solubility or poor permeability before synthesis, accelerating the identification of orally viable candidates [109]. CYP liability prediction is similarly essential: pkCSM, admetSAR, and FAME 3 help flag structural alerts for CYP3A4/CYP2C19 inhibition or rapid metabolic turnover. A JAK screening project applied pkCSM to remove chemotypes predicted to strongly inhibit CYP3A4 while prioritizing balanced clearance profiles [95]. Cardiotoxicity filtering—especially hERG inhibition—has become equally important. Recent JAK3 covalent inhibitor campaigns integrated ProTox-II and CardioTox models to exclude electrophiles or aromatic motifs associated with QT prolongation [109]. Multi-parameter optimization (MPO) is widely used to balance potency, permeability, solubility, and stability; in TYK2/JAK1 dual-inhibitor efforts, MPO-based filtering improved hit progression and correlated strongly with enzymatic and cellular assay success [108]. Virtual ADMET-driven library triaging—using SwissADME, pkCSM, and ProTox-II— enables pre-screening of thousands of derivatives for oral drug-likeness, GI absorption, CYP inhibition risk, mitochondrial toxicity, and hepatotoxicity [109], Several recent JAK discovery studies reported eliminating >60% of poor-quality candidates before synthesis, significantly improving efficiency [[11], [12], [13]].

## 8 Conclusion and future perspectives

The rapid expansion of Janus kinase (JAK) inhibitor research reflects the central role of the JAK–STAT pathway in immune regulation, hematopoiesis, inflammation, and oncogenic signalling. Since the first demonstrations of JAK2 inhibition in leukemia cells, clinically approved agents—including ruxolitinib, tofacitinib, baricitinib, fedratinib, and upadacitinib—have transformed the management of autoimmune, inflammatory, and myeloproliferative diseases. However, limited isoform selectivity, off-target kinase interactions, dose-limiting toxicities, and acquired resistance continue to constrain long-term efficacy. These challenges highlight the need for next-generation JAK inhibitors with enhanced precision, safety, and pharmacokinetic performance. Advances in structural biology have refined our understanding of JAK2 and JAK3 architecture, revealing new opportunities for rational selectivity engineering through hinge-region optimization, pseudokinase (JH2) modulation, and covalent engagement of the uniquely druggable Cys909 residue in JAK3. Parallel medicinal-chemistry innovations—such as heterocyclic scaffold design, C–H activation, photoredox catalysis, multicomponent reactions, and fragment-based discovery—have accelerated the generation of potent, fine-tuned scaffolds. These synthetic developments synergize with computational methodologies including structure-based design, molecular docking, molecular dynamics, QM/MM calculations, free-energy perturbation, deep-learning QSAR, and generative AI for *de novo* ligand design. Together, these approaches increase binding predictability, streamline lead optimization, and reduce attrition. Looking ahead, the future of JAK inhibitor development will depend on overcoming persistent challenges in isoform discrimination, toxicity, and resistance. Allosteric and pseudokinase-domain inhibitors may offer alternatives to classical ATP-competitive mechanisms. Covalent and reversible-covalent JAK3 inhibitors targeting Cys909 represent a promising strategy for durable, highly selective therapies. Multi-target or hybrid inhibitors may better address complex inflammatory or oncogenic networks.

An emerging and highly promising extension of next-generation JAK therapeutics is the development of JAK-targeting protein degraders, particularly proteolysis-targeting chimeras (PROTACs). Structure-guided design of JAK degraders has demonstrated that selective depletion of JAK2 or JAK3 can be achieved by coupling high-affinity JAK-binding warheads with optimized E3 ligase ligands, enabling catalytic target removal rather than transient inhibition. Early preclinical studies of JAK-directed PROTACs report sustained pathway suppression, improved durability of response, and the potential to overcome resistance mechanisms associated with kinase reactivation. Continued advances in linker engineering, ternary complex stabilization, and isoform-selective warhead optimization are expected to further expand the therapeutic scope of JAK degraders in inflammatory and oncologic indications. Looking forward, several concrete directions are poised to shape the next phase of JAK inhibitor discovery. Beyond ATP-competitive paradigms, the identification and validation of novel allosteric sites within regulatory domains, including the pseudokinase (JH2) region and interdomain interfaces, offer opportunities to achieve durable pathway modulation with reduced on-target toxicity. Covalent and reversible-covalent strategies targeting non-conserved residues, when combined with precise control of warhead reactivity and residence time, may further enhance isoform selectivity and safety. In parallel, structure-guided development of JAK-targeting protein degraders, including PROTAC-based strategies, is emerging as a complementary approach to achieve sustained pathway suppression through catalytic degradation rather than transient kinase inhibition. In parallel, AI-driven drug design is expected to transition from retrospective optimization toward prospective, patient-aware discovery, integrating genomic, transcriptomic, and pharmacokinetic data to guide personalized inhibitor selection. Together, these approaches highlight a shift from broadly acting kinase inhibition toward precision-engineered JAK therapeutics tailored to disease biology and individual risk profiles. Finally, AI-driven modelling, digital retrosynthesis, automated high-throughput screening, and advanced structural prediction are poised to reshape early discovery and accelerate translation into clinical agents.

## CRediT authorship contribution statement

**Karthik K. Karunakar:** Conceptualization. **Binoy Varghese Cheriyan:** Data curation. **Sowmiya Philiph:** Conceptualization. **Rajesh kumar Shanmugam:** Data curation, Conceptualization. **Josme Sree:** Conceptualization.

## Ethics approval

Not applicable.

## Declaration of generative AI in scientific writing

During the preparation of this work, the authors used QuillBot and Paperpal to improve readability and language. After using these tools, the authors reviewed and edited the content as needed and takes full responsibility for the content of the publication.

## Funding information

Not Applicable

## Conflict of interest statement

I am the corresponding author of the manuscript titled **“Next-Generation Janus Kinase Inhibitors: Integrating Synthetic Innovation, Structural Biology, and Computational Design for Precision Drug Discovery”** hereby declare that we have **no conflicts of interest** related to the work presented in this article.

No author has any financial, personal, or institutional relationships that could be perceived as influencing the research described in this manuscript. We further confirm that the study was conducted independently, without any commercial or financial support that could introduce bias.

All authors have read and approved this statement and agree to its submission along with the manuscript.

## Data Availability

No data was used for the research described in the article.
